# Editorial: The role of natural products in neurological disorders

**DOI:** 10.3389/fneur.2023.1163282

**Published:** 2023-04-13

**Authors:** Magda Tsolaki, Chrisa Arcan, Saadia Zahid

**Affiliations:** ^1^Alzheimer Hellas and Laboratory of Neurodegenerative Diseases, Center for Interdisciplinary Research and Innovation (CIRI-AUTh), Aristotle University of Thessaloniki, Thessaloniki, Greece; ^2^Epidemiology Division, Department of Family Medicine and Population Health, Virginia Commonwealth University Health System, Richmond, VA, United States; ^3^Department of Healthcare Biotechnology, Atta-ur-Rahman School of Applied Biosciences (ASAB), National University of Sciences and Technology (NUST), Islamabad, Pakistan

**Keywords:** natural products, Mediterranean diet (MD), extra virgin olive oil (EVOO), brain health, Alzheimer's disease

Neurological disorders constitute over 6% of the global burden of disease. Unfortunately, many neurological diseases, such as stroke, Alzheimer's, Parkinson's, multiple sclerosis, migraine, and amyotrophic lateral sclerosis, have no cure. Depression, migraine, and dementia are neuropsychiatric diseases that strongly correlate with the functional status of brain.

Twenty-five centuries ago, the father of medicine, Hippocrates, coined the famous quote “Our diet is our medicine, and our medicine is our diet.” Being true to this philosophy, Hippocrates and other Greek physicians used diet as the cornerstone of their medical practice. Owing to the rich content of bioactive compounds, natural or plant-based products have demonstrated their potential health benefits in improving symptoms of different diseases. However, only a small number of plants have been investigated for neurological or neuropsychiatric disorders. Increasing evidence has shown that natural products are linked to the improvement of various disease biomarkers. More importantly, these products are perceived as safer alternatives to pharmacotherapy, with lower risk of adverse effects or withdrawal symptoms. In addition to natural products, various dietary patterns have shown great promise in lowering the risk of many chronic diseases.

There has been a recent editorial about the use of natural products for the management of anxiety and depression (1). Data from the World Health Organization (WHO) has indicated that 4.5% of global population suffers from major depressive disorder (MDD), placing it as one of the most common psychiatric disorders that is posing a serious public health challenge (2). The first course for the management of depression is medications, but they are effective among only 50% of patients, provide a delayed response, and have multiple side effects. Taking this into consideration, the editorial highlighted the effectiveness of six different natural products observed either in animal models or in human randomized clinical trials (1).

In this collection of articles, two papers focused on the effects of traditional Chinese medicine (TCM). TCM, through mediation of the gut microbiota, exerts anti-depressant-like effects, providing evidence that it is safe and effective in the treatment of depression (3). Perhaps the gut microbiota may be the key mediator in the link between natural products and neuropsychiatric diseases. The study by Zhao et al. focused on the mechanisms responsible for the reduction of a depression-like state in rats by specifically testing two isomers, albiflorin (AF) and paeoniflorin (PF), extracted from the root of *Paeoniae Radix* Alba, belonging to the monoterpene glycosides. The mechanism may have linked with the regulation of the neuroendocrine immune system and disrupted the metabolic pathways. This provides a new perspective for cancer-related depression therapy (Zhao et al.). These actions are also related to the gut microbiota.

According to a systematic analysis by the Global Burden of Disease Study, in 2016 migraine affected 1.04 billion people and caused 45.1 million years of life lived with disability globally (4). There are no medications to date that can reduce the symptoms or prevent the onset of migraine without side effects. Lyu et al., who in their retrospective hospital-based study analyzed the electronic medical records of patients who were diagnosed with migraines, found that Chinese herbal medicine was the predominant treatment in 88.5% of patients with reported significant improvements in migraine symptoms. These findings suggest that Chinese herbal medicine is worth being evaluated in randomized controlled trials as an alternative to conventional pharmacotherapies (Lyu et al.).

Iron accumulation in the brain leads to neurological disorders. Interestingly, several phytochemicals such as flavones and polyphenols are effective metal chelators and have been shown to decrease iron-induced neurotoxicity. The third article of this topic by Alikhani et al. investigated the chelating capacity of ferulic acid and caffeic acid in the brain tissues of iron-overload mice. The study demonstrated that both ferulic acid and caffeic acid significantly decreased iron content in the brain and serum, suggesting that these natural compounds might be plausible natural iron chelators for brain iron-overload therapy (Alikhani et al.).

A recent review described the multiple bioactive constituents of saffron (*Crocus sativus* L.) and their consistent beneficial effects against a range of human neuropsychiatric disorders (depression, anxiety, sleeping alterations, etc.) (5). In a human trial by Tsolaki et al., daily intake of 125 mg of saffron for one year resulted in significant improvements in neuropsychological exams, volumes of MRI, and event-related potentials in patients with mild cognitive impairment (MCI) (6).

The complexity of the pathogenesis of Alzheimer's disease (AD), the most frequent form of dementia, is the main reason for the repeated failure of anti-AD drugs. Among the six FDA-approved drugs, one is a natural product—galantamine—while recently approved sodium oligomannate capsules (GV-971) in China demonstrate the potential of natural products in the treatment of neurodegenerative diseases. Therefore, the focus of AD treatment strategies is gradually shifting to therapies involving natural products. Improving dietary intake, such as selecting a Mediterranean dietary pattern (Medi) or an Asian diet rich in fruits and vegetables, is globally recognized as an effective means to prevent the development of AD. These mainly plant-based diets are rich in polyphenols, commonly found in extra virgin olive oil (EVOO), vegetables, and fruits such as grapes, blueberries, and apples, in addition to tea and red wine. Polyphenols have diverse and complex structures and are mostly found in glycosidic forms in plants (7). The main food group in Medi is EVOO. Tsolaki et al. conducted a three-arm randomized controlled trial in 50 participants with MCI. The participants were given either early harvest extra virgin olive oil (EHEVOO), Medi+ EHEVOO, or only Medi. After 12 months, the Medi+ EHEVOO group experienced better cognitive abilities compared to Medi alone, especially in general cognition, attention, and fluency tasks, independently of the presence of the APOEε4 gene (8, 9). Vassilopoulou et al., using a confirmatory factor analysis, examined the dietary components of the MIND diet among Greek elderly populations. The study found that 9 specific components of the MIND diet score, compared to the original 15 items, were more effective in discriminating dementia patients from healthy controls, indicating that the short version can be used in Greek elderly groups (Vassilopoulou et al.).

## Author contributions

MT drafter the editorial. CA critically edited and formatted. SZ critically edited. All authors contributed to the article and approved the submitted version.
